# The Structure Characteristics of Laminar Premixed Flames of Gasoline-like Fuel Under CI Engine-Relevant Conditions

**DOI:** 10.1021/acsomega.4c00895

**Published:** 2024-06-08

**Authors:** Yuanyuan Zhao, Zongyu Yue, Yan Zhang, Chenchen Wang, Yuqing Cai, Yong Chen, Zunqing Zheng, Hu Wang, Mingfa Yao

**Affiliations:** † State Key Laboratory of Engines, Tianjin University, Tianjin 300350, PR China; ‡ CAEP Software Center for High Performance Numerical Simulation, Beijing 100088, China; § Institute of Applied Physics and Computational Mathematics, Beijing 100088, China

## Abstract

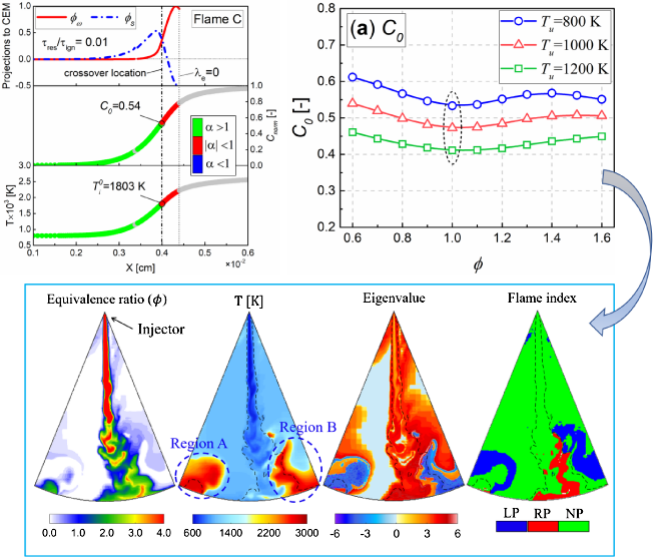

Gasoline compression ignition characterized by partially premixed and long ignition delays typically features complex flame structures such as deflagration or spontaneous ignition fronts. In this study, the flame structure and propagation characteristics of PRF90/air mixtures under compression ignition engine-relevant conditions are investigated numerically. Similar to other types of fuels, under such conditions, the propagation speed of PRF90 laminar premixed flames depends not only on the unburnt mixture properties but also on the residence time, and the transition of the flame regime depends only on the residence time. Nevertheless, due to the temperature-dependent autoignition chemistry of PRF90, flames with excessively high unburnt temperatures show different combustion behaviors after the transition from deflagration to autoignition-assisted flames. Sensitivity analysis showed that, the dominant chain branching reactions in the deflagration mode are H + O_2_ = OH + O and CO + OH = CO_2_ + H, and that in the autoignition-assisted flames with lower unburnt temperature are H_2_O_2_(+M) = 2OH(+M) and IC_8_H_18_ + HO_2_ = AC_8_H_17_ + H_2_O_2_, while for higher unburnt temperatures, the reactions C_3_H_5_ + HO_2_ = C_2_H_3_ + CH_2_O + OH and IC_8_H_18_ = IC_4_H_9_ + TC_4_H_9_ are more important than the fuel low-temperature oxidation reactions. In addition, a criterion based on chemical explosive mode analysis is used to analyze the local combustion mode. The results show that the difference in diffusion/chemical structure at the crossover progress variables *C*_0_ and crossover temperature  allows both *C*_0_ and  to be used as a flame location for distinguishing propagation modes in premixed flame. However, the effects of the equivalence ratio on *C*_0_ are different from that on , which means that the selection of *C*_0_ and  may lead to different discriminant results for stratified mixtures. Comparing the applicability of *C*_0_-based and -based locations in three-dimensional gasoline compression ignition flame, it is found that the flame location based on the value of *C*_0_ at ϕ = 1.0 can more completely reflect the flame development characteristics in stratified premixed combustion.

## 1 Introduction

Currently, most practical compression ignition (CI) engines are diesel engines using conventional diesel fuel, in which the spray combustion process of diesel fuel inevitably generates high levels of engine-out soot and nitrogen oxides (NOx). In the past decade, the use of gasoline-like fuels instead of diesel fuel in CI engines is considered to be one of the most promising ways to achieve efficient and clean combustion, which is called gasoline compression ignition (GCI).^1−3^ By employing gasoline-like low reactivity fuels, ignition delay is prolonged, allowing the fuel to be partially mixed with air to form a stratified mixture at elevated temperatures and pressures prior to ignition, thereby reducing the particulates and NOx emissions. Under these conditions, deflagration or spontaneous ignition fronts, or combination of both, could exist depending on the competition between the residence time of unburnt mixture (τ_res_) and the corresponding ignition delay time (τ_ign_).^4−6^ A number of studies have been shown that the varied flame regimes have great impacts on the flame propagation and heat release characteristics of the combustion process.^7−9^ Therefore, understanding the flame propagation and structure characteristics of gasoline-like fuel under CI engine-relevant conditions is of practical importance for optimizing the combustion process.

Studies have shown that the laminar flame speed *S*_l_ of premixed fuel/air mixture is a key parameter for understanding the transition of flame propagation and structure characteristics.^10−12^ In the past few decades, many scholars have measured the laminar flame speed of gasoline-like fuels under nonignitioned conditions (approximately 0.1–2.5 MPa and 320–600 K),^13−17^ which provides a basis for the characterization and modeling of gasoline combustion and the design of practical combustion devices such as gasoline internal combustion engines. However, it is worth noting that CI engines operate at elevated pressures and temperatures (more than 1000 K). Under such elevated thermodynamic conditions, experiments on laminar premixed flame propagation can hardly be performed and therefore numerical simulations are often used.^18^ In addition, under such elevated thermodynamic conditions, *S*_l_ depends not only on the thermochemical properties of the unburnt fuel/air mixture, but also on the induction length (*L*) and τ_res_.^19^ For example, Habisreuther et al.^18^ and Sankaran^20^ found that at higher temperatures, *S*_l_ increased with increasing *L*, resulting in an eventual change in a flame regime from the canonical deflagration wave to spontaneous ignition front. Gong and Ren^5^ demonstrated the relationship between *S*_l_ and τ_res_ based on the calculation of one-dimensional (1D) freely propagating laminar premixed flames of *n*-heptane/air at elevated temperatures and pressures and pointed out that the flame structure is further complicated by the two-stage ignition processes associated with the negative temperature coefficient behavior. However, there is scarce literature on the laminar flame speed and flame structure characteristics of gasoline-like fuels at elevated temperatures and pressures.

In addition, as has been previously noted, both autoignition and deflagration could coexist due to the mixture stratification in GCI during the combustion process, and it is critical to identify the different flame propagation modes and to capture the processes controlling the flame propagation. In order to systematically describe the flame structure of different propagation modes, Xu et al.^21^ recently proposed a quantitative diagnostic method based on chemical explosive mode analysis (CEMA) to distinguish different local combustion modes. Specifically, with this approach, local combustion modes can be determined by quantifying the relative importance of projected chemical (ϕ_ω_) and diffusion (ϕ_s_) source terms in the pre-ignition mixture. In addition, due to the differences in local combustion modes between deflagration and autoignition, the ratio of ϕ_s_/ϕ_ω_ at crossover temperature  was further used to determine flame regimes. At present, this CEMA-based method has been applied to distinguish the flame propagation mode for premixed combustion.^21−23^ However, GCI is typical of mixture stratified combustion, in which the crossover temperature  can vary with local equivalence ratio ϕ and fresh mixture temperature. Therefore, the applicability of the criterion conditioned on *T =* to mixture stratified combustion (e.g., GCI) still needs to be further explored. In contrast, the normalized reaction process variable is widely used to characterize the thermochemical space in various combustion configurations as it is independent of mixture temperature and equivalence ratio. Jaravel et al.^24^ investigated the relationship between ϕ_s_*/*ϕ_ω_ and process variable in one-dimensional laminar flames and found that the flame propagation regime of fast flame and detonation can be readily distinguished using the Xu’s criterion at low progress variable. However, the aforementioned studies mainly focus on full premixing mixtures rather than stratified mixtures.

The aim of this study was first to understand the flame structure characteristics of laminar premixed flame of gasoline-like fuel (e.g., primary reference fuel) under engine-relevant elevated thermodynamic environments. Second, the effect of equivalence ratios on the crossover location was studied to explore whether there exists a suitable flame location that can be used to distinguish flame propagation regimes in stratified premixed mixtures. In order to achieve the research objectives of this work, the normalized propagation speed and the normalized residence time were used to reveal the factors affecting the flame regime transition, sensitivity analysis of flame propagation speed was used to identify the controlling chemistry in flame propagation, and the local combustion mode analysis based on CEMA was used to describe the diffusion/chemical structure.

## 2 Methodology

### 2.1 Modeling and Analytical Approach

**Figure 1:**
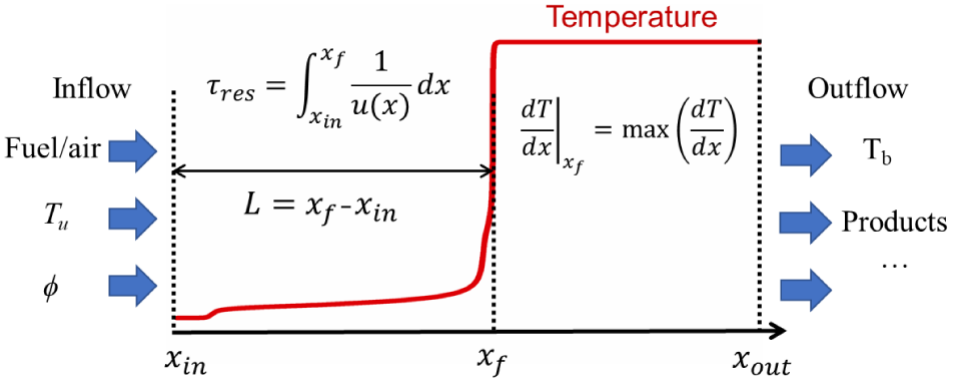
Schematic of the inflow–outflow domain of a 1D freely propagating premixed flame.

In current study, 1D freely propagating laminar premixed flame simulations under different inlet conditions were performed using Cantera et al.^25^ A schematic of the 1D Cartesian inflow-outflow computational domain from *x*_in_ to *x*_out_ is shown in Figure 1. At the inlet of the domain, the reactant composition, equivalence ratio (ϕ), and unburnt temperature (*T*_u_) are prescribed with a hard inflow boundary condition. For freely propagating flames, ambient pressure (*p*) is a constant, and *S*_l_ is an eigenvalue solution corresponding to the inlet velocity. In the calculations, the distance from the inlet boundary *x*_in_ to the flame location *x*_f_ identified by the maximum temperature rise rate is defined as the induction length *L*. The residence time for the unburnt mixture (τ_res_) is defined as
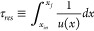
1where *u*(*x*) is the local velocity of the fluid mixture. The ignition delay time, τ_ign_, is defined as the time interval from the start of the calculation to the maximum temperature increase rate in constant-pressure 0D autoignition.

**Figure 2:**
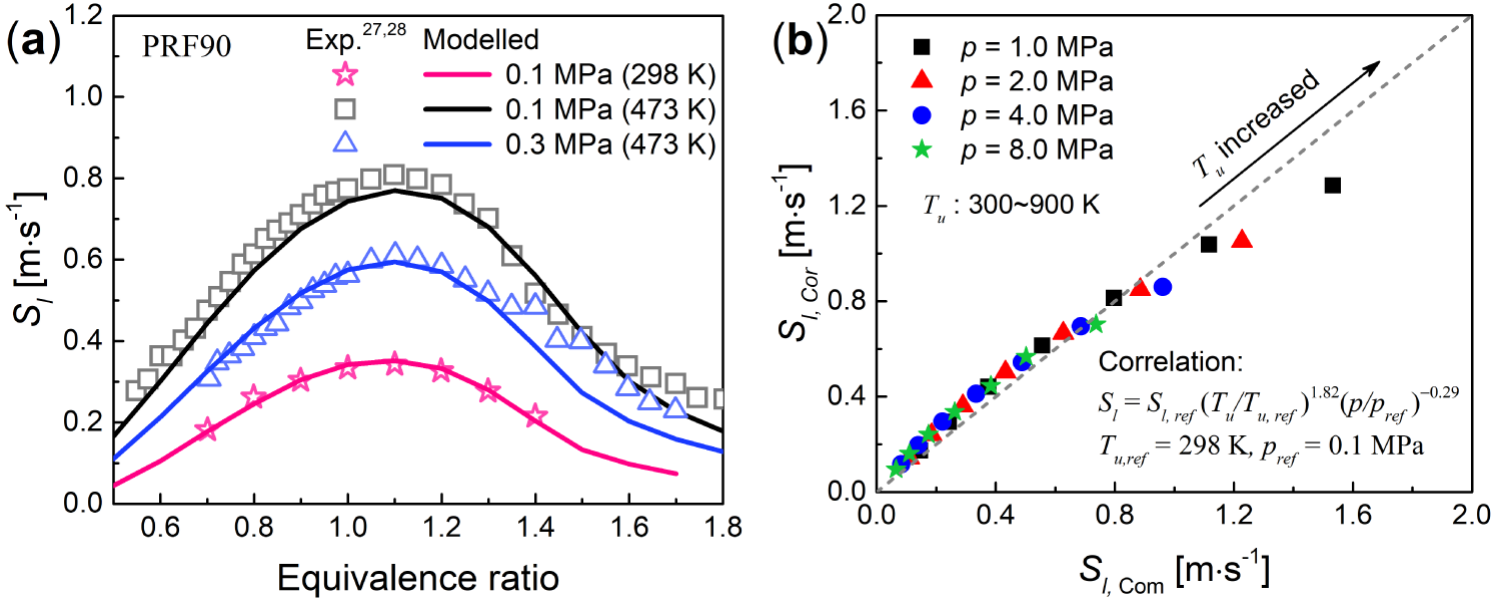
Validations of the reduced PRF mechanism with laminar burning velocity at elevated temperatures and pressures. (a) Compared with *S*_l_ measured by experiments^27,28^ and (b) compared with theoretical correlation.

In this work, the primary reference fuel 90 (PRF90, 90% iso-octane and 10% *n*-heptane by volume) was considered as gasoline-like fuel. The reduced PRF mechanism with 73 species and 296 reactions is used and validated against the ignition delay time measured experimentally at higher pressures (e.g., 4.0 MPa).^26^ Meanwhile, Figure 2a shows that the calculated *S*_l_ for PRF90 are in excellent agreement with the experiments^27,28^ at 0.1 and 0.3 MPa. Unfortunately, the laminar flame velocities of PRF90/air mixtures at higher pressures, such as above 4.0 MPa, have not yet been measured experimentally, which is also beyond the scope of this study. A fitting formula on laminar flame velocity,^13,29^*S*_l_ = *S*_l,ref_(*T*_u_/*T*_u,ref_)^α^(*p*/*p*_ref_)^β^, has been derived by calculating the temperature exponent (α) and pressure exponent (β) from the measured data in Figure 2a. The theoretical correlation for PRF90 was used to further validate the predicted laminar burning velocity at both elevated temperatures and elevated pressures. It is seen from Figure 2b that the predicted laminar burning velocities of the reduced PRF mechanism agree very well with the computed ones by the correlation equation.

In this study, the flame structure and propagation speed of freely propagating PRF90/air flames are numerically investigated over the range of unburnt temperatures of 500–1300 K, an ambient pressure of 4.0–7.0 MPa, and an equivalence ratio of 0.6–1.6. To ensure grid independent solutions, the mesh was adaptively refined so that the slope and curvature of the solution were less than 0.02 and 0.05, respectively.

### 2.2 Local Combustion Mode Indicator Based on CEMA

A CEMA-based method by Xu et al.^21^ is applied to distinguish different local combustion modes. The CEMA^30^ is a systematic flame diagnostic based on eigen-analysis of the Jacobian of the local chemical reaction source term (***J***_ω_) in the governing equation of a reaction-diffusion flow:
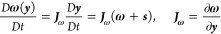
2where **ω** is the chemical source term and ***s*** is nonchemical source terms, such as diffusion in flames and homogeneous mixing in stirred reactors. The parameter λ_e_ was defined as the eigenvalue of ***J***_ω_. The chemical explosive mode (CEM) associated with a positive λ_e_ reveals the propensity of an isolated local mixture to ignite. CEM is present in pre-ignition mixtures and disappears postignition. Projecting both chemical and diffusion terms onto the left eigenvector ***b***_e_ associated with CEM leads to
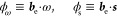
3where the scalars ϕ_ω_ and ϕ_s_ are the projection of the chemical and diffusion terms, respectively. Then, the ratio
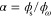
4can be used to compare the relative contributions of diffusion and chemistry source terms in an ignition process and describes three different local combustion modes: (i) α > 1: a local diffusion-assisted ignition mode where diffusion plays a dominant role and facilitates the consequent ignition process.; (ii) |α| < 1: a local autoignition mode where chemistry dominates diffusion; (iii) α ← 1: a local extinction mode where the diffusion dominates but works against the chemical reaction process.

Although the mode indicator α conditioned on the crossover temperature  is chosen to distinguish different local flame propagation modes in premixed flames,^21−23,31^ the application of  in stratified premixed conditions has yet to be validated. On the other hand, considering that the normalized reaction process variable is independent of mixture temperature and equivalence ratio, a crossover progress variable (*C*_0_) determined by the combination of a normalized reaction progress variable and a laminar premixed flame structure is introduced here. In the present study, a species-based progress variable (*Y*_c_) proposed by Lucchini et al.^32^ is chosen to describe the whole combustion process.
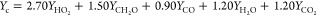
5

Since *Y*_c_ is dependent on mixture fraction *Z*, a normalized progress variable *C*_norm_ is defined as
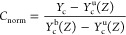
6where  and  are the minimum and maximum values of *Y*_c_ at a given mixture fraction, respectively.^33,34^

## 3 Results and Discussion

### 3.1 Freely Propagating PRF90/Air Flames

**Figure 3:**
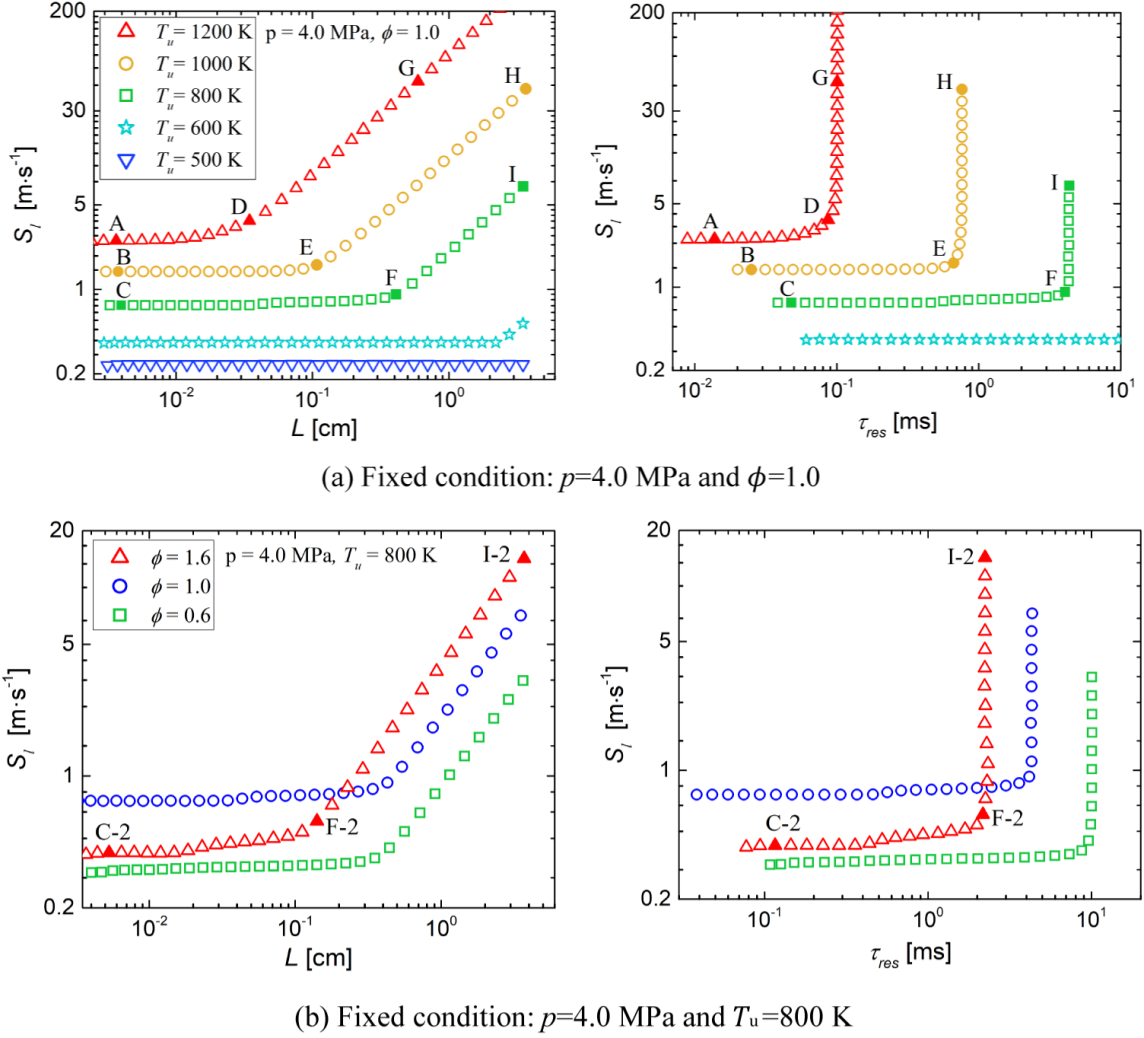
Variation of the laminar flame speed with the induction length and residence time for 1D freely propagating premixed flames at different inlet conditions.

Figure 3a demonstrates the computed flame propagation speed *S*_l_ as a function of the induction length *L* and the residence time τ_res_ for PRF90/air flames at unburnt temperatures of 500–1200 K, an equivalence ratio of 1.0, and a pressure of 4.0 MPa. It is shown that for each unburnt temperature, the profiles feature a plateau for small *L*, where the propagation speed is approximately a constant and is denoted as the reference flame speed , which increases with unburnt temperature. Similar to the observation for hydrogen^21^ and *n*-heptane,^5^ with continually increasing *L* from the unique shorter *L*, an approximate linear correlation between *S*_l_ and *L* is observed, with the slope inversely proportional to the ignition delay time of the inlet mixture. On the other hand, the correlation between *S*_l_ and τ_res_ shows that as the residence time becomes sufficient for the mixture to autoignite when entering the reaction front, there is a sharp increase in *S*_l_, generally indicating the transition from a canonical deflagration wave to an autoigniting wave at the turning point.

In addition, it is worth noting that, as shown in Figure 3b, as the equivalence ratio increases from 0.6 to 1.6, the flame propagation speed curves cross due to the nonmonotonic dependence of  on the equivalence ratio, despite the fact that the autoignition delay time monotonically increases with the equivalence ratio. Similarly, the nonmonotonic dependence of  on the equivalence ratio can be seen in Figure 2a. That is, compared to the rich mixture, the stoichiometric mixture may require a longer induction length to reach a certain propagation speed, although the stoichiometric mixture has a higher reference flame speed. Additionally, it can be inferred from Figure 3b that under such elevated temperature and pressure conditions, even for the lean premixed mixture, a long residence time will lead to too fast a flame propagation speed, which is unfavorable to the combustion control of the engine. On the other hand, combining Figure 3a,b, it can be observed that the reference flame speed seems to be independent of induction length and the residence time but is determined by the unburnt mixture properties.

**Figure 4:**
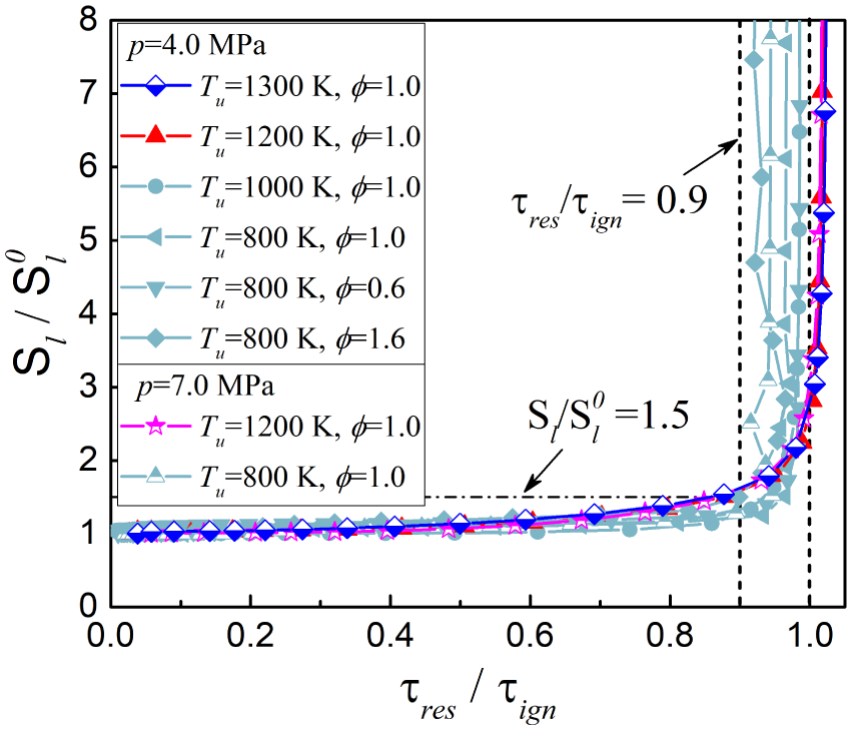
Normalized flame speed *S*_l_/ against the normalized residence time τ_res_/τ_ign_ for PRF90/air flames at different inlet conditions.

Figure 4 shows the normalized flame speed *S*_l_/ against the normalized residence time τ_res_*/*τ_ign_. According to Krisman et al.,^19^ small values of τ_res_/τ_ign_ in general mean that autoignition is unimportant, values near or above unity indicate that autoignition is likely to occur, and intermediate values may imply a combination of deflagration and autoignition. As shown, for all curves, in the range of τ_res_/τ_ign_ < 0.9, the value of *S*_l_/ did not exceed 1.5, and the propagation speed tends to be constant as the residence time decreases. Besides, when τ_res_/τ_ign_ > 0.9, the propagation speed shifts and increases sharply, suggesting that residence time uniquely determines the transition from deflagration to autoignition, which is similar to the findings with natural gas flame^19^ and *n*-heptane flame.^5^ On the other hand, we notice that for *T*_u_ ≥ 1200 K, after the transition, the value of τ_res_/τ_ign_ slightly exceeds the unity, while it is less than the unity in all other cases with *T*_u_ < 1200 K, which imply that excessively high unburned temperatures affect the combustion behavior in autoignition mode.

### 3.2 Sensitivity Analyses for Flame Propagation Speed

To further identify the controlling chemistry in flame propagation, sensitivity analysis of flame propagation speed for different flame regimes is investigated based on the flames selected from Figure 3, which are detailed in Table 1. It is seen that flames A–C2 in Group 1 correspond to smaller *L* and τ_res_/τ_ign_, flames G–I2 in Group 3 correspond to larger *L* and τ_res_/τ_ign_, and flames D–F2 in Group 2 are near the turning points.

**Table 1:** Details of the Flames are Selected from Figure 3^a^

flame		*T*_u_ (K)	ϕ	τ_res_/τ_ign_	*L* (cm)	*S*_l_ (m·s^–1^)
group 1	A	1200	1.0	0.14	0.004	2.525
	B	1000	1.0	0.03	0.004	1.402
	C	800	1.0	0.01	0.004	0.738
	C-2	800	1.6	0.05	0.005	0.391
group 2	D	1200	1.0	0.88	0.035	3.681
	E	1000	1.0	0.86	0.108	1.594
	F	800	1.0	0.90	0.412	0.893
	F-2	800	1.6	0.89	0.141	0.576
group 3	G	1200	1.0	1.02	0.596	52.547
	H	1000	1.0	0.99	3.674	45.471
	I	800	1.0	0.97	3.519	7.102
	I-2	800	1.6	0.93	3.709	14.245

a All flames in the table have a pressure of 4.0 MPa.

**Figure 5:**
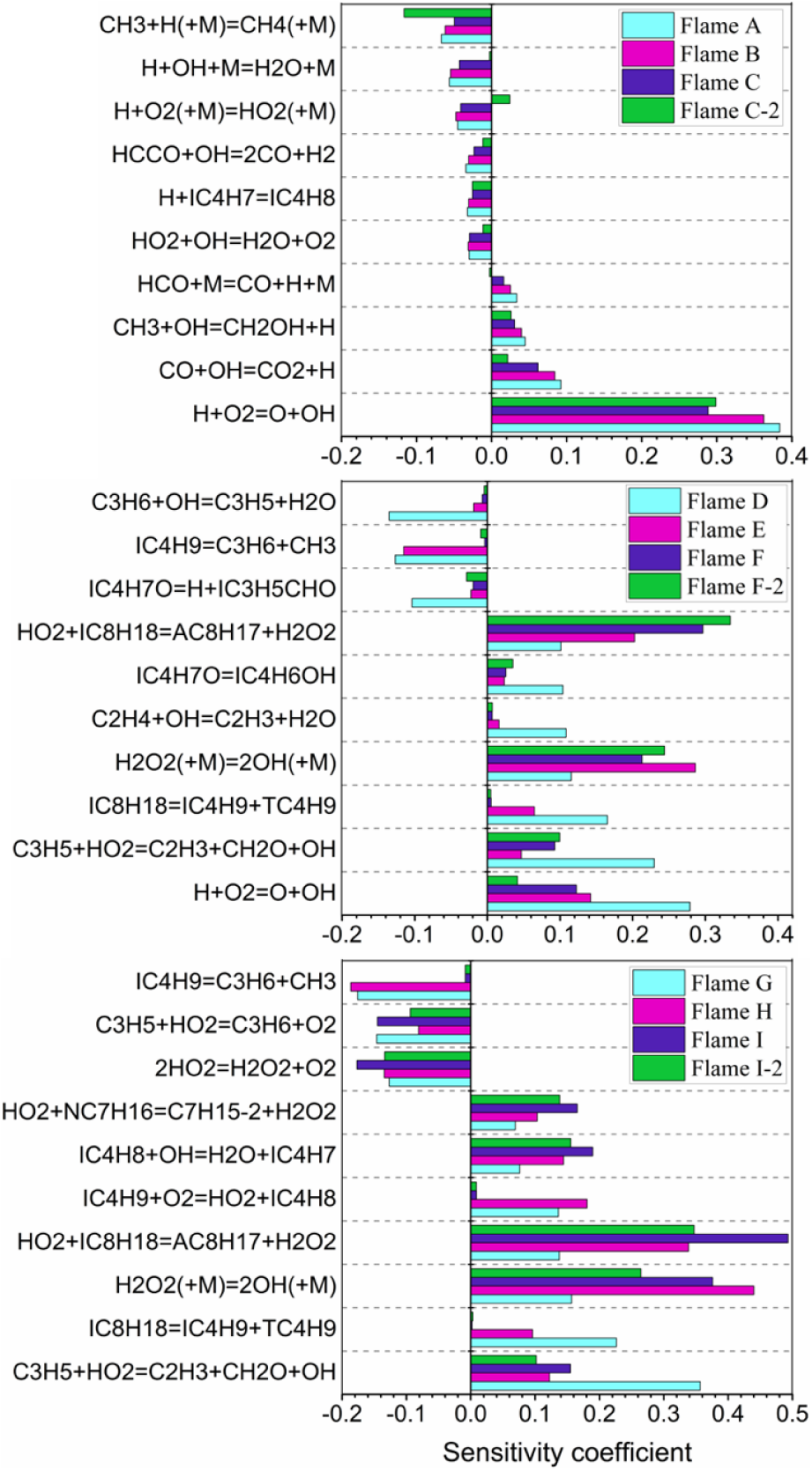
Sensitivity analyses of the flame propagation speed for the flames listed in Table 1.

As shown in Figure 5, it is observed that for flames in Group 1, the most important reactions are the chain branching reaction H + O_2_ = OH + O and chain propagation reaction CO + OH = CO_2_ + H, which is consistent with the existing understanding of flame chemistry under normal thermodynamic conditions.^5,35,36^ Compared with the flames in Group 1, the flames in Group 2 and Group 3 showed significant changes. For example, for flame D, the dominant chain branching reaction remains H + O_2_ = O + OH, while the role of CO + OH = CO_2_ + H does not even make it into the top 10. For flame G, the dominant chain branching reaction transforms into C_3_H_5_ + HO_2_ = C_2_H_3_ + CH_2_O + OH, followed by IC_8_H_18_ = IC_4_H_9_ + TC_4_H_9_, and then H_2_O_2_(+M) = 2OH(+M). Unlike flames D and G, the dominant chain branching reactions for flames with lower unburnt temperatures, such as E, F, F-2, H, I, and I-2, involve H_2_O_2_(+M) = 2OH(+M) and the first dehydrogenation of the fuel, e.g., IC_8_H_18_ + HO_2_ = AC_8_H_17_ + H_2_O_2_, implying the controlling role of autoignition chemistry. From the above analyses, it is clear that the impacts of autoignition chemistry on flame propagation are enhanced with increasing *L* and τ_res_ under elevated thermodynamic conditions relevant to internal combustion engine operation and that the autoignition chemistry of PRF fuels is strongly influenced by unburnt temperature.

### 3.3 Flame Structure Under Different Flame Propagation Modes

**Figure 6:**
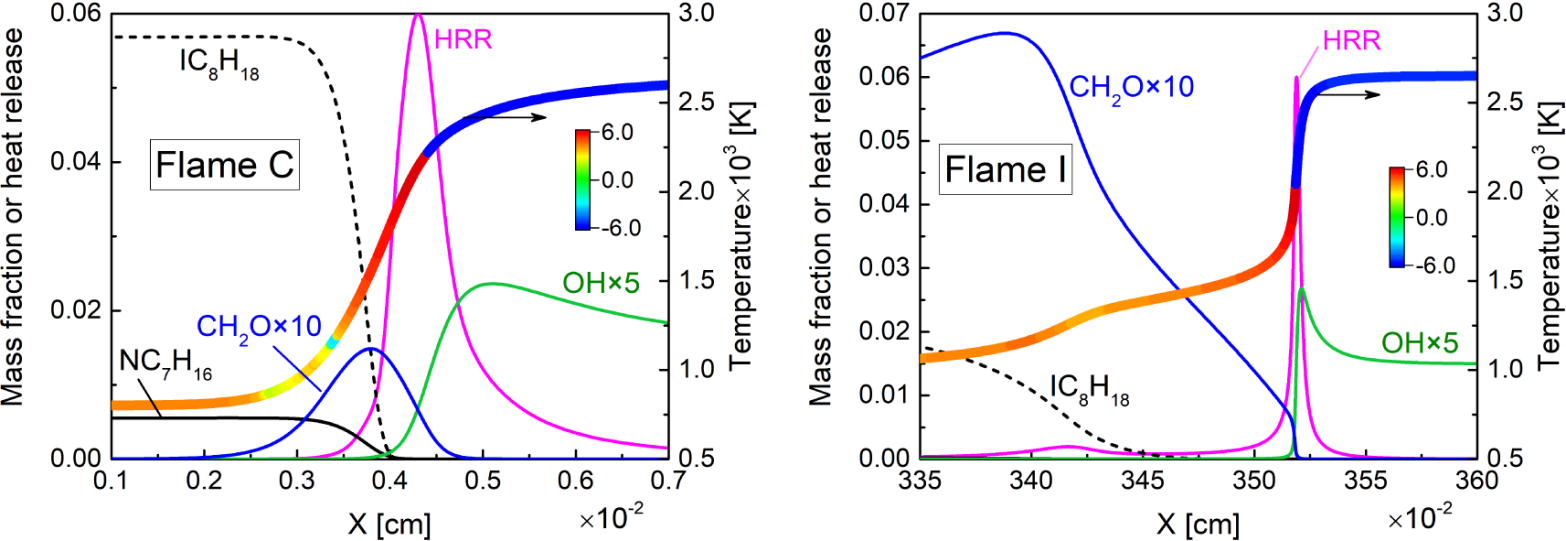
Profiles of temperature, mass fractions of IC_8_H_18_, NC_7_H_16_, CH_2_O, and OH, and normalized heat release rate for flames C and I. The heat release rate (HRR) profile shows the value of . Color on the temperature profile indicates the value of sign (λ_e_) × log_10_(1 + |λ_e_|, s^–1^).

To compare the flame structure under different propagation regimes, Figure 6 shows the temperature profiles with colors indicating the value of CEM eigenvalue, the profiles of normalized heat release rate, and mass fractions of key species for the different types of flames. It is seen that for the two selected flames here, the mixture becomes explosive with increasing temperature as the mixture approaches the flame zone, and the λ_e_ value peaks near the location of the maximum heat release rate. Hydroxyl radical (OH), known as a marker of the reaction zone, peaks after the zero-crossing of λ_e_. Furthermore, it can be found that formaldehyde (CH_2_O), known as a marker of the preheat zone, peaks prior to the zero-crossing of λ_e_, and its peak location gradually moves away from the reaction zone as the propagation regimes transforms to autoigniting wave. In addition, a clear two-stage heat release process is observed in flame I, which corroborates the effect of the low-temperature fuel oxidation on the flame propagation in the low-unburnt-temperature flame in the previous sensitivity analyses.

**Figure 7:**
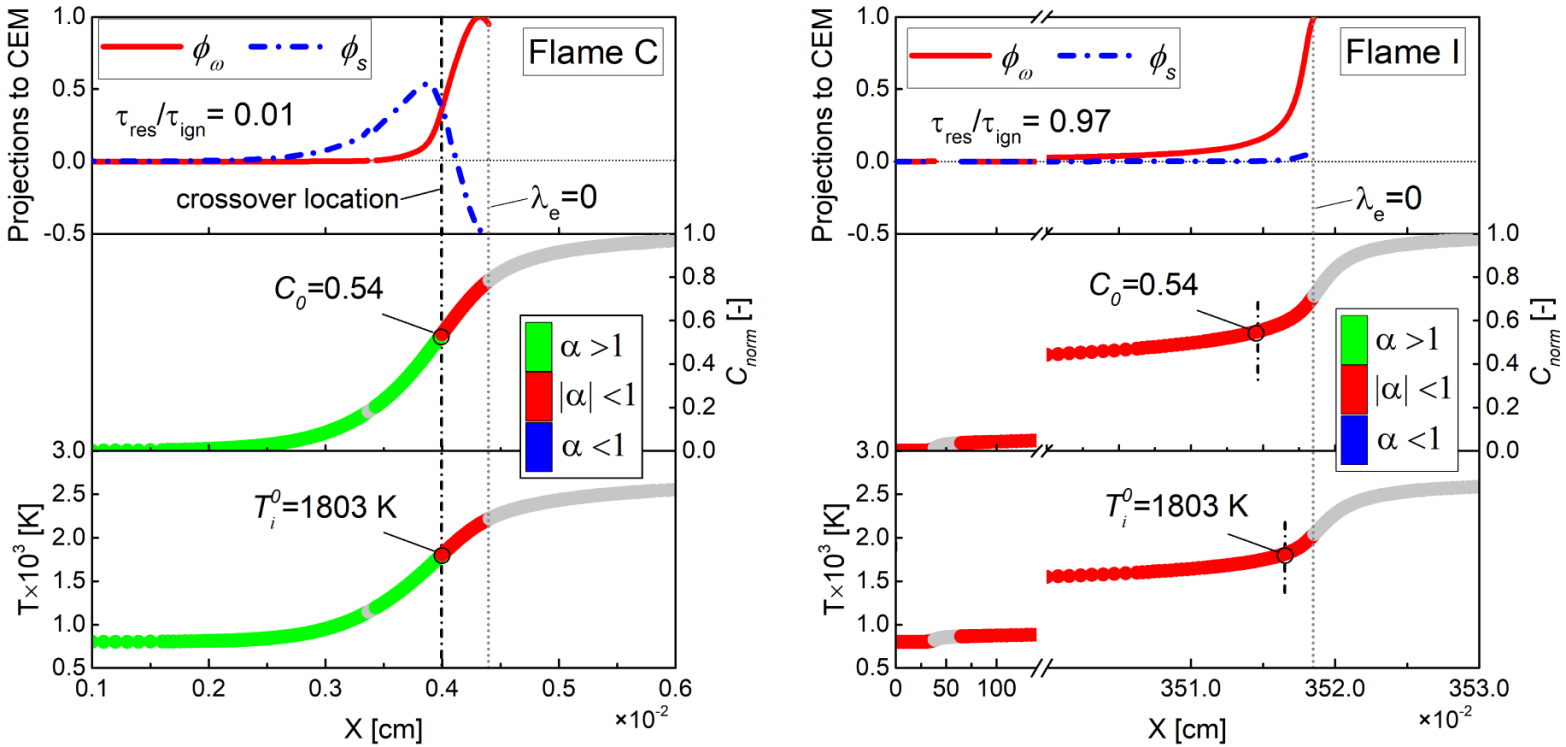
Profiles of ϕ_ω_, ϕ_s_, *C*_norm_ and temperature for flames C and I. Color on the progress variable and temperature profiles indicate the assisted-ignition mode (green), autoignition mode (red), extinction mode (blue), and nonexplosive mixtures (λ_e_ < 0, gray).

Figure 7 shows the distribution of the local combustion modes overlaid on the progress variable profile and the projected chemical (ϕ_ω_) and diffusion (ϕ_s_) source terms for the different types of flames at the two unburnt temperatures. Colors on the profiles of progress variable and temperature indicate local modes in the pre-ignition mixtures (λ_e_ > 0). Note that the only difference between the boundary conditions of flames C and I is that the τ_res_/τ_ign_ of the former is much lower than that of the latter. As shown, for each unburnt temperature, the diffusion effect is diminishing from flame C to flame I, suggesting a transition to autoignition mode. On the other hand, it is observed that flame C is diffusion-dominated (α > 1) throughout the preheating zone of *C*_norm_ < *C*_0_ (or ) and chemically dominated (|α| < 1) within the next thin layer of *C*_norm_ > *C*_0_ (or ). For flame I as autoigniting wave, |α| < 1 is seen throughout the pre-ignition zone. Further comparing flames C and I, it can be found that the diffusion-chemical structures of flames C and I at the cross location are completely different, which means that the α value at the crossover location (*C*_norm_ = *C*_0_ or ) obtained from the deflagration wave (e.g., flame C) can be used to determine the propagation type of the flame front under the same thermodynamic conditions (i.e., *T*_u_, *p*, ϕ). The above analysis of local combustion modes for different propagation types of flames is similar to that for hydrogen flames,^21^ except that the latter was described on the basis of the crossover temperature  corresponding to the intersection of ϕ_ω_ and ϕ_s_.

**Figure 8:**
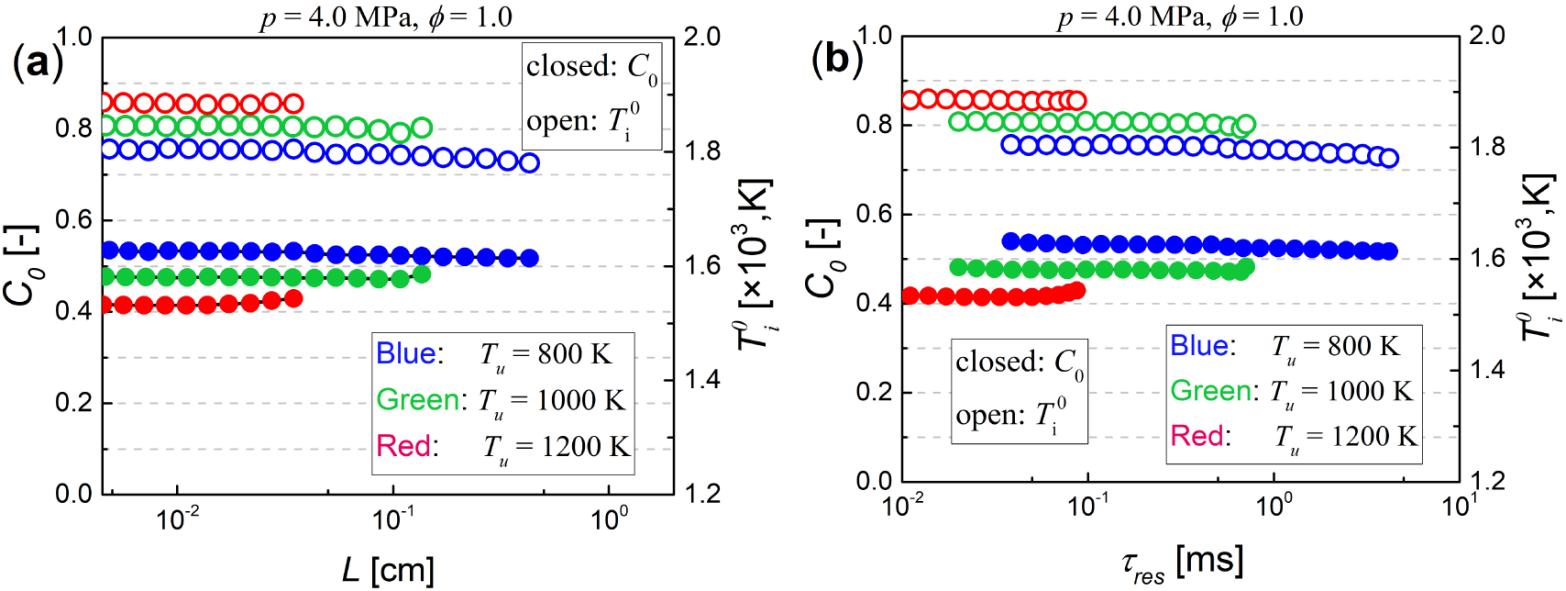
Crossover location *C*_0_ and , as a function of (a) *L* and (b) τ_res_, for the flames with *S*_l_/<1.5 simulated in Figure 3a.

Figure 8 further examined the sensitivity of the crossover location *C*_0_ and  for flames with *S*_l_/<1.5 (without autoigniting wave) to induction length and residence time. It is seen that, for the mixture with defined inlet conditions, *C*_0_ and  are approximately constant at smaller *L* and τ_res_, while near the turning point *C*_0_ rises slightly and  decreases slightly. Overall, the effect of induction length and residence time on the crossover location in such flames with *S*_l_/<1.5 is almost negligible.

### Influence of Fuel Stratification on *C*_0_ and 

3.4

Considering that the GCI engine is typically operated at partial premixed combustion and characterized by the mixture with equivalence ratio stratification in the cylinder ranging from fuel-rich (>1.2) to fuel-lean (<0.8) at the onset of ignition,^37,38^ it is necessary to investigate the effect of equivalence ratio on the crossover location *C*_0_ and . To ensure that the flame was a deflagration wave, the induction length was fixed to a smaller value of 0.006 cm in the following content.

**Figure 9:**
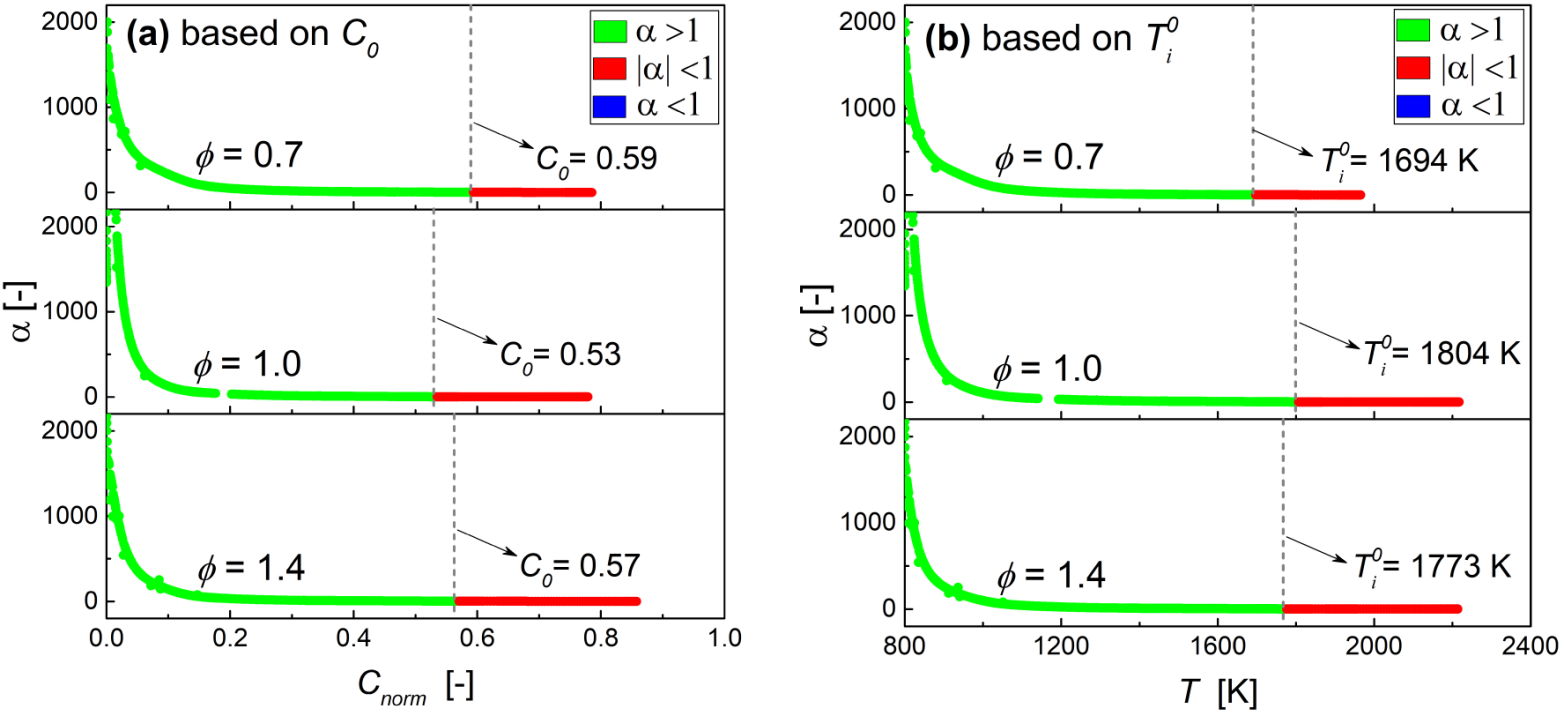
Profile of α vs (a) *C*_norm_ and (b) temperature for PRF90/air 1D freely propagating premixed deflagration waves at different equivalence ratio for the conditions of *T*_u_ = 800 K, *p* = 4.0 MPa, and *L* = 0.006 cm. The vertical dashed line corresponds to the crossover location (*C*_0_ or ) of each flame. The nonexplosive region with λ_e_ < 0 is truncated.

Figure 9 shows the distribution of α on *C*_norm_ and the temperature in the deflagration wave under three different equivalence ratios. It is seen that for the three equivalence ratios of 0.7, 1.0 and 1.4, *C*_0_ is the minimum while  is the maximum in the case of stoichiometric mixture, which indicates that the influence of the equivalence ratio on *C*_0_ and  is completely different. Interestingly, a lower *C*_0_ or  may be used as an unambiguous flame location for combustion mode identification in such three equivalence ratios mixture. For example, as shown in Figure 9, the α values at *C*_0_= 0.53 or at =1694 K for the three equivalence ratios are greater than unity.

**Figure 10:**
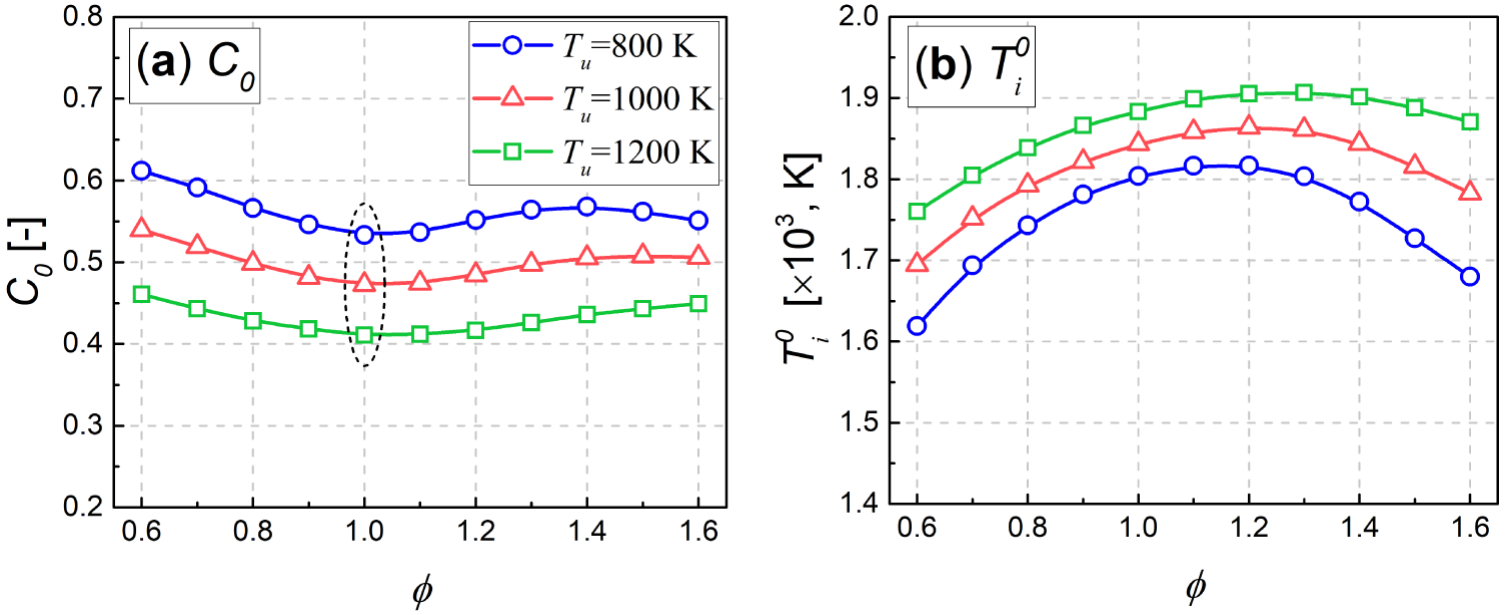
Crossover location based on (a) *C*_0_ and (b) , as a function of equivalence ratio ϕ, for the deflagration waves at *p* = 4.0 MPa and *L* = 0.006 cm.

Figure 10 further demonstrates the crossover location (*C*_0_ and ) versus equivalence ratio for the three unburnt temperatures. As shown, for each unburnt temperature, the *C*_0_ decreases and then increases with increasing equivalence ratio in the range of equivalence ratios of 0.6–1.6, while  shows a roughly opposite trend to *C*_0_. In addition, it can be found that *C*_0_ has a minimum value near ϕ = 1.0, while a lower value of  could be obtained theoretically only in fuel-lean or fuel-rich mixtures. From the relationship between  and the equivalence ratio shown in Figure 10(b), it can be inferred that the equivalence ratio used to obtain  could not be an unambiguous value, especially for stratified premixed mixtures characterized by lean-and-rich premixed.

**Figure 11:**
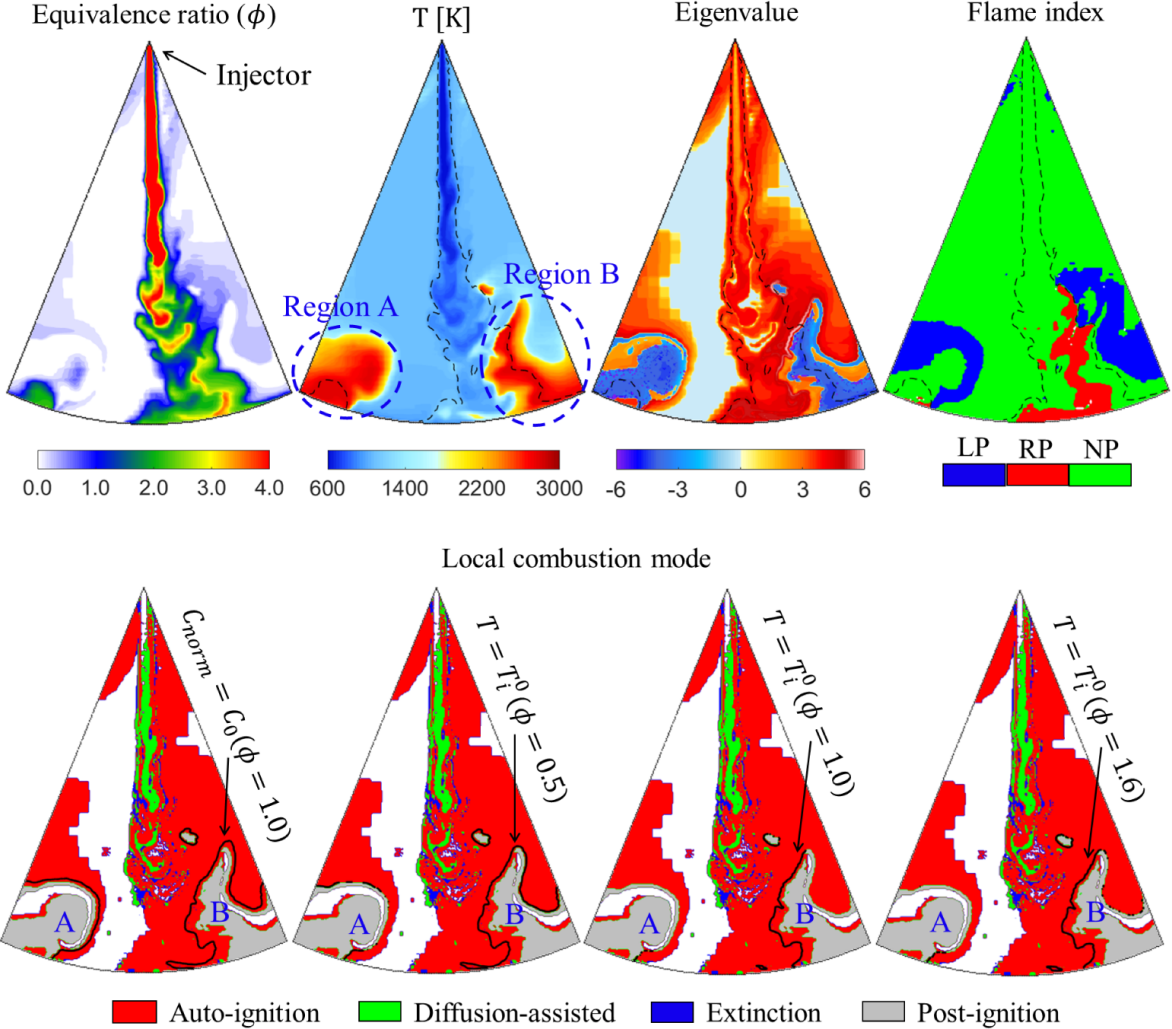
Distributions of the equivalence ratio, temperature, eigenvalue, flame index, and local combustion mode in GCI combustion at 3.1° CA ATDC. Color in eigenvalue fields indicates sign(λ_e_) × log_10_ (1 + |λ_e_|, s^–1^). Colors on the flame index plots indicate the lean-premixed (blue, “LP”), rich-premixed (red, “RP”), and nonpremixed (green, “NP”) states, respectively.

To compare the applicability of *C*_0_-based and -based crossover locations in three-dimensional (3D) GCI flames, Figure 11 shows the distribution of the equivalence ratio, temperature, CEM eigenvalue, flame index, and the local combustion modes for GCI combustion fueled with PRF90 at the early stage of flame development. The simulation results of the 3D flame here have been fully validated against the experimental data.^39^ Note that the flame index, , is a parameter that determines premixed/nonpremixed flame based on the scalar product of the gradients of the fuel (∇*Y*_F_) and oxidizer  mixture fraction fields,^40,41^ where *Z*_st_ is the stoichiometric mixture fraction. In addition, in the local combustion mode plots, the chemically inactive zone, cold flame (λ_e_ < 0 and lower temperature), and the gas–liquid two-phase zone near the nozzle are truncated because they are not involved in this study. The inlet conditions of 1D freely propagating premixed flames to determine the crossover location are close to that of the in-cylinder thermodynamic conditions near the onset of ignition. Under such inlet conditions, the *C*_0_ at an inlet equivalence ratio (ϕ) of 1.0 is 0.48, while the values of  under three inlet equivalence ratios (0.5, 1.0, and 1.6) are 1632, 1905, and 1806 K, respectively. Note that the black dashed lines in the temperature, eigenvalue, and flame index plots are the stoichiometric mixture fraction contours.

As shown in Figure 11, the high-temperature flame zones, Region A and Region B, are mainly located downstream of the spray, and these zones not only have the characteristics of stratified mixture but also contain three states of lean premixed, rich premixed, and nonpremixed. The distribution of flame index also shows that the 3D GCI flame is typical of partially premixed combustion characteristics. In addition, the distribution of local combustion modes shows that the high-temperature flame zone mainly corresponds to the postignition (λ_e_ < 0) mode, and outside the postignition mode is the autoignition mode and the diffusion-assisted mode (near the spray core), as well as the extinction mode.

The solid black lines in the local combustion modes show the spatial distribution of *C*_0_ at inlet equivalence ratio (ϕ) of 1.0 and  at equivalence ratios of 0.5, 1.0, and 1.6, respectively. Since the crossover location should meet the premise of λ_e_ > 0,^21^ the parts of λ_e_ < 0 in the isolines of *C*_0_ and  are truncated for convenience of comparison. Obviously, only the isolines of *C*_0_ at ϕ = 1.0 completely enclose Region A and Region B, and are spatially continuous, while the isolines of  under the three equivalence ratios are incomplete, especially the part of the isolines of  in the lean premixed regime is missing. This observation shows that, compared with the crossover location based on , the crossover location based on the value of *C*_0_ at ϕ = 1.0 can well capture the flame propagation process of both lean premixed and rich premixed combustion, and can be used as an unambiguous flame location for combustion mode identification in stratified premixed combustion. Based on such characteristics of *C*_0_ at ϕ = 1.0, we conducted a numerical study on the in-cylinder ignition and flame development process of GCI in another work^39^ and found that both spontaneous ignition and deflagration wave existed in GCI flames, while the former dominated the flame propagation process.

## 4 Conclusions

A numerical and theoretical study for 1D freely propagating premixed gasoline-like fuel/air flames under CI engine-relevant conditions was conducted to investigate flame structure characteristics when the flame regime transition occurs. PRF90 is chosen as the fuel, and a reduced PRF fuel mechanism is considered. A CEMA-based criterion and sensitivity analysis were employed to analyze local combustion modes and the controlling chemistry in flame propagation for different flame regimes, respectively. The main conclusions are

(1) Under CI engine-relevant conditions, the propagation speed of PRF90 laminar premixed flames depends not only on the unburnt mixture properties but also on the residence time. The transition of the flame regime from deflagration to autoignition, meanwhile, is determined only by the residence time, which is in general agreement with the findings for other types of fuels such as *n*-heptane,^5^ hydrogen,^20^ and natural gas.^19^
(2) The normalized residence time analysis shows that after the flame regime transition, in contrast to the case of other unburnt temperatures, the normalized values τ_res_/τ_ign_ of PRF90/air mixtures with an excessively high unburnt temperature slightly exceed the unity. Further sensitivity analyses showed that the controlling chemistry in flame propagation varies depending on the flame regime and is strongly influenced by the unburnt temperature. Specifically, in the deflagration wave, the dominant chain branching reactions in flame propagation are H + O_2_ = OH + O and CO + OH = CO_2_ + H. When the flame regime evolves toward the autoignition mode, the reactions H_2_O_2_(+M) = 2OH(+M) and IC_8_H_18_ + HO_2_ = AC_8_H_17_ + H_2_O_2_ dominate at *T*_u_ < 1200 K, while for *T*_u_ ≥ 1200 K, the reactions are, in order of importance, C_3_H_5_ + HO_2_ = C_2_H_3_ + CH_2_O + OH, IC_8_H_18_ = IC_4_H_9_ + TC_4_H_9_ and then H_2_O_2_(+M) = 2OH(+M).
(3) On the other hand, the local combustion mode analysis showed that when the flame regime transitions from deflagration to autoignition, the preheating zone prior to the crossover location (*C*_0_ and ) transformed from diffusion dominated to chemically dominated. The difference in diffusion/chemical structure in different flame regimes allows the crossover location *C*_0_ and  to be used as a flame location for distinguishing flame regimes in premixed combustion. In addition, the crossover location *C*_0_ and  are negligibly affected by the induction length and residence time.
(4) Nevertheless, there are significant differences in the effects of equivalence ratio on *C*_0_ and . The value of *C*_0_ decreases and then increases with increasing equivalence ratio in the range of equivalence ratios of 0.6–1.6, while  shows a roughly opposite trend to *C*_0_. The comparison of the applicability of the *C*_0_-based and -based crossover locations in 3D GCI flame shows that the flame location based on the value of *C*_0_ at ϕ = 1.0 can more completely reflect the flame development characteristics in stratified premixed combustion.
